# Detecting Blood Flow Response to Stimulation of the Human Eye

**DOI:** 10.1155/2015/121973

**Published:** 2015-10-04

**Authors:** Alex D. Pechauer, David Huang, Yali Jia

**Affiliations:** Casey Eye Institute, Oregon Health & Science University, Portland, OR 97239, USA

## Abstract

Retinal blood supply is tightly regulated under a variety of hemodynamic considerations in order to satisfy a high metabolic need and maintain both vessel structure and function. Simulation of the human eye can induce hemodynamics alterations, and attempt to assess the vascular reactivity response has been well documented in the scientific literature. Advancements in noninvasive imaging technologies have led to the characterization of magnitude and time course in retinal blood flow response to stimuli. This allowed for a better understanding of the mechanism in which blood flow is regulated, as well as identifying functional impairments in the diseased eye. Clinically, the ability to detect retinal blood flow reactivity during stimulation of the eye offers potential for the detection, differentiation, and diagnosis of diseases.

## 1. Introduction

Vascular tissue in the human eye is unique in that it can be directly and noninvasively observed in vivo. The clinical importance of assessing retinal structure and vascular function has been a fundamental part of ophthalmology since the origins of the ophthalmoscope [1]. Since this time, impairment of retinal circulation has been identified as having a key role in the pathogenesis of some of the leading causes of blindness, including macular degeneration and diabetic retinopathy [2–5]. Vascular deficits that constitute the pathophysiology of these diseases have been extensively studied but are still not well understood.

Complexity in retinal tissue structure and function makes it one of the most metabolically active locations in the body [6]. Blood supply to the retina must be tightly regulated under various hemodynamic considerations [7, 8]. Localized effects such as changes in intraocular pressure and light stimulation, as well as systemic effects such as altered blood gas concentration and fluctuations in blood pressure, have all been found to cause significant changes in blood flood or vessel resistance [5]. Vascular reactivity in response to stimulation of the human eye has been characterized in both the healthy and diseased eye [9, 10]. Although the relationship and interactions of the influences on retinal blood flow are numerous and complex, a marked impairment in vascular reactivity during these hemodynamic alterations has been consistently observed in the diseased eye [11]. Clinically, the ability to assess retinal blood flow during stimulation of the eye offers potential for the detection, differentiation, and diagnosis of diseases. Furthermore, the severity of impaired vascular reactivity can be used to monitor disease progression or evaluate treatment [11].

Noninvasive imaging techniques have led to new and important information regarding vascular reactivity. Advancements in technology have allowed for better measurement sensitivity and reliability in determining the effects of various types of eye stimulation. The purpose of this review is to describe the various imaging techniques and measurements used to investigate vascular reactivity, compare the different methods of human eye stimulation, and discuss the application in the diseased eye.

## 2. Vessel Diameter

### 2.1. Fundus Photography

Since the invention of the ophthalmoscope, vessel caliber has been used to assess retinal vessel health [1, 12, 13]. In 1916, Moore observed arteriosclerosis of the retina and suggested that an overall decrease in the size of retinal arteries was an early sign of the disease [13]. Studies using the ophthalmoscope were qualitative, and it was not until the invention of fundus photography in 1931 that researchers were able to directly measure the diameters of retinal vessels [14]. With this method, repeated images of the eye were captured and magnified with a low power (9x) microscope [15]. Sieker and Hickam used this technique to measure diameters of retinal vessels ranging between 90 and 180 microns in size [15]. In an effort to increase measurement sensitivity, a sophisticated micrometric technique was developed that projected and magnified (35x) fundus images on a translucent screen. An operator then positioned a thin wire along the vessel edge which allowed for computer calculation of diameter [16]. Higher resolution fundus photos and repeated measurements taken during systole have improved sensitivity to vessels approximately 20 microns in size [17]. Because of this relatively large size of observable retinal vessel, diameter measurements using fundus photography are limited to 0.5 to 1.5 disc diameters away from the optic disc margin [18].

Fundus photography is one of the earliest methods used to measure vascular reactivity in response to stimulation of the eye. Since diameter change is only indirectly related to the change in blood flow, it provides a qualitative index into vascular reactivity. By measuring percent change in diameter, Sieker and Hickam were able to demonstrate vasoconstriction of retinal vessels during changes in blood gas concentration [15]. They showed that, in a healthy population, breathing 100% pure oxygen resulted in an 11.5% decrease and a 14.0% decrease in arterial and venous caliber, respectively. This constriction had a tendency to decrease with an increase in age. Furthermore, they showed a marked impairment in hyperoxic response in diabetic participants with complications such as retinopathy, neuropathy, and hypertension [15].

Vascular reactivity in response to alterations in breathable carbon dioxide did not have such a clearly defined effect. Huerkamp and Rittinghaus reported vasodilation following inhalation of increased carbon dioxide [19], while Hickam et al. found no substantial dilation during inhalation of mixtures containing either 5% or 10% carbon dioxide [20]. In order to investigate this discrepancy, vessel diameter and blood oxygen concentration were both measured during changes in breathable carbon dioxide and oxygen concentrations [21, 22]. It was found that oxygen is more effective in changing vessel diameter compared to carbon dioxide. However, 10% carbon dioxide in mixtures containing either 21% or 90% oxygen caused a significant increase in retinal venous oxygen saturation, despite causing no significant change in vessel diameter. This suggests that vascular reactivity had occurred in vessels that were below the minimum vessel size for fundus photography [21].

More recently, fundus photography has been used to measure diameter changes in response to light stimulation of the retina. One minute of continuous flickering light caused a small but significant increase in arterial and venous diameter [17]. This was a short response, and after cessation of light, vessel diameter returned to preflicker levels within 6 seconds. Light stimulation is localized and does not have the lasting effects or large response caused by a systemic change in blood gas. Unlike the response to oxygen, arteries were found to have a greater response compared to veins of similar size. On average the difference in diameter size was 4.2 ± 2.2% (mean ± SD) for arteries and 2.7%  ± 1.7% for veins [17]. Although significant, these results are relatively close to the limitations of measureable change using fundus photography.

Diameter measurements using fundus photography are limited by the operator's ability to accurately delineate the vessel edge. One report found the intertrial variance among three images graded by the same observer to be less than 5% for both the microscopic and projection method [23]. Furthermore, care must be given to ensure repeated diameter measurements are taken at precisely the same location. Repeatability in this manner is dependent on the operator's experience [16]. Interobserver difference was found to be more significant, as large as 11% [24]. Considering this, fundus photography may be unable to accurately detect vessel caliber. Measuring percent change in diameter during stimulation of the eye can attenuate these limitations and provide a reliable application for measuring vessel diameter using fundus photography [24]. However, given the system noise, it may be dangerous to identify deficits in vascular reactivity in the diseased eye using this technique.

### 2.2. Fundus Video

**Figure 1:**
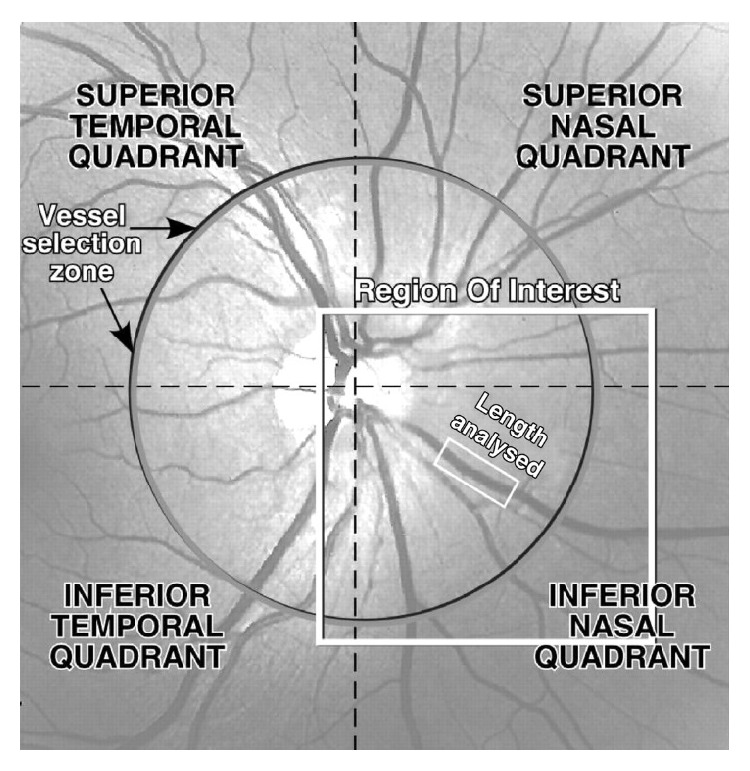
Fundus image as seen on the RVA monitor. Rectangular white box is the length of vessel measured (reprinted from [25] Copyright Association for Research in Vision and Ophthalmology).

Advances in technology allowed fundus photography to be adapted for continuous computer analysis with the retinal vessel analyzer (RVA). The RVA uses a fundus video camera and computer algorithm to obtain real time measurements of retinal artery and vein diameters. This combines high temporal and spatial resolution allowing for reproducible calculations of diameter in vessel sections (Figure 1) [25] about 1.5 mm in length and 20 microns in width [25–27].

Fundus video analysis using RVA was most impactful with light stimulation experiments, allowing researchers to measure the rapid and small changes in vessel diameter [28]. Previously, using fundus photography, researchers were unable to precisely photograph and measure the maximum response of retinal vessel. Using RVA, Polak et al. describe a method of averaging video diameter measurements taken during the last 20 seconds of a 60-second flicker stimulation [29]. This had the added benefit of reducing the effect of pulsatility. Kotliar et al. improved on this method by identifying a maximum vessel dilation after only 10 seconds of flicker [28]. Averaged diameter measurements during the seven seconds around this maximum were calculated to account for systolic and diastolic fluctuations. This resulted in diameter changes of the same or greater magnitude than those recorded by Polak, despite measurements being taken after a shorter flicker duration [28, 29]. However, comparison between these two experiments is dangerous due to the difference in design and method.

In addition to the maximum response, RVA also allowed for the characterization of a dynamic response of retinal vessels to light flicker stimulation. Exposure to 12 Hz of flickering light caused an immediate dilation of arteries. Retinal veins also dilated, but this response was delayed about 5 seconds. Both arteries and veins then continued to dilate until a “saturation point,” after which they would experience regulation and begin to constrict. For arteries, saturation occurred before cessation of flicker. Veins, however, were shown to reach this point later, either at or after cessation [28]. Arteries, but not veins, continued to constrict past the original baseline diameter, demonstrating an “overshot” regulatory response. This finding is different from that of Polk, who found both the arteries and veins experienced a dilation overshoot of 80.6% and 57.9% original diameter, respectively [29].

Deficits in flicker response have been reported in diabetic subjects with no or nonproliferative retinopathy. Investigations of insulin dependent diabetics showed no significant dilation in either retinal arteries or veins during 64 seconds of light flicker. In the same experiment, healthy controls experienced a maximum response of 2.8% (SD 2.2%) increase in retinal arterial diameter [30]. This response is similar in magnitude to measurements made using fundus photography [17]. Other studies of healthy eyes found that induced hyperglycemia reduces the flicker-induced vasodilation in vessels by 55% [31]. In both of these studies, it is unclear whether this is because of a diminished retinal reactivity or a decrease in neural activity [30]. However, these findings do suggest the relevance of blood flow control in diabetes related eye diseases.

The main limitation of determining vascular reactivity with both traditional fundus photography and fundus video using RVA is that only the diameter of large vessels, adjacent to the optic disc, can be measured. Since vessel reactivity increases with decreasing vessel size, it is possible that much of the blood flow control is occurring in vessels below the threshold of this technology [21, 32]. Furthermore, diameter measurements only detect vascular resistance and do not allow for direct detection of blood flow.

## 3. Fluorescein Angiography

**Figure 2:**
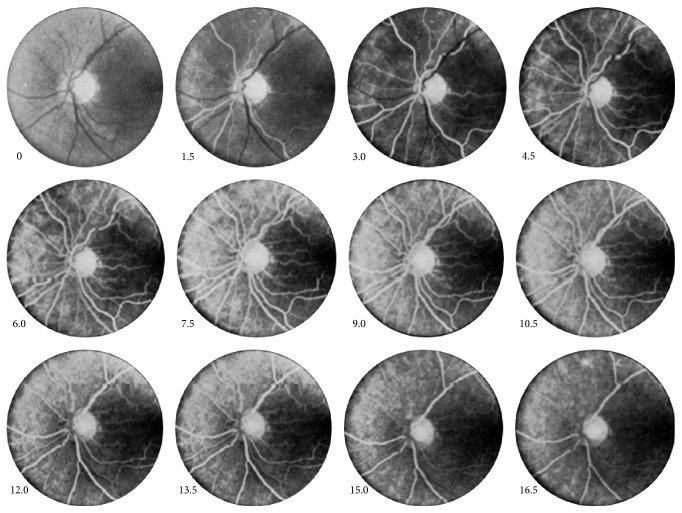
Serial photographs, taken every 1.5 seconds, showing the passage of fluorescein through the retinal circulation in a normal eye. Photography marked “0” is the last in series before the appearance of fluorescein. Sequence of pictures shows the arteries filled with fluorescein followed by veins (reprinted from [34]).

**Figure 3:**
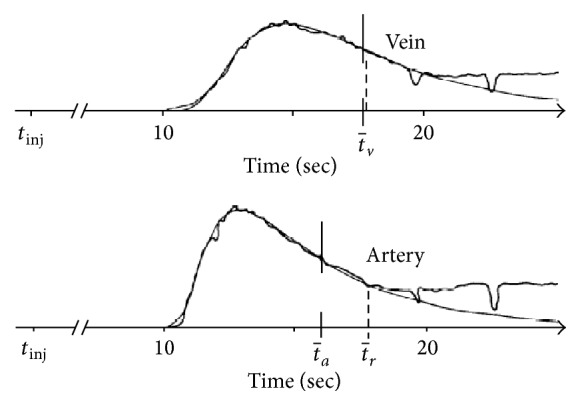
Typical fluorescence-intensity curves recorded from superior temporal segment of retina in a healthy subject with no ocular abnormalities. Both curves begin at approximately the same time. *t*_*r*_ is the time at which recirculation begins and *t*_*a*_ and *t*_*v*_ are mean times of fluorescein from site of injection to site of measurements along artery and vein, respectively. Smooth curves are log-normal distribution functions that optimally fit arterial and venous first passages (reprinted from [36]).

The dye dilution technique (DDT) provides a retinal circulation flow index by determining the time it takes for fluorescein to enter and clear a vessel segment. Before visualization, an injection of the fluorescent dye is made into the antecubital vein of the arm [33]. Serial fundus photos are then taken to determine the intensity of fluorescence during illumination with blue light at 490 nm (Figure 2) [34]. For each picture, density measurements estimate the relative concentration of the dye in both the retinal arteries and veins [34, 35]. Density measurements are then used to create fluorescence-intensity curves (Figure 3) [36]. The mean circulation time (MCT) is then calculated as the difference between the mean venous and arterial passage times. The advent of fundus video allowed for continuous analysis of density measurements and more accurate MCT calculations [37–39]. Another DDT approach utilizes a scanning laser ophthalmoscope (SLO) to determine the arteriovenous passage (AVP) time [40, 41]. This is accomplished by calculating the time difference between when the dye first appears in an artery and when it first appears in the adjacent vein. The fluorescein velocity can be found using SLO by calculating the difference in appearance time between two points a known distance apart [42, 43]. A study using SLO to measure AVP time and mean arterial dye velocity determined the variations of these parameters in a population of 221 healthy participants. Within-participant coefficient of variation (CV) was 15.6% for the arteriovenous passage and 16.7% for the mean arterial dye velocity. The between-participant CV was slightly higher at 20.7% for arteriovenous passage time and 23.7% for the mean arterial dye velocity [44].

Hickam and Frayser used the fluorescein DDT to establish a mean circulation time of 4.7 ± 1.1 seconds in 29 healthy men [35]. They also demonstrated that, during 100% oxygen inhalation, MCT was prolonged and retinal blood flow decreased to 57% of its initial rate [34]. Studies using SLO also suggest that changes in blood flow in the retina are paralleled by matched changes in oxygen content [41]. This is thought to maintain a relatively constant oxygen delivery rate despite a fluctuating blood oxygen concentration. In diabetic patients, hyperoxia did not prolong AVP, suggesting regulatory dysfunction and uncontrolled oxygen delivery rates [43].

Although fluorescein angiography is a standard imaging and diagnostic technique in clinical ophthalmology, quantification of vascular reactivity with the DDT is difficult in the diseased eye. In the eye, DDT provides only some index of mean blood flow through a close vascular segment. Vascular abnormalities such as occlusion, neovascularization, and vessel leakage can affect the flow time [33, 45]. Therefore, it is impossible to determine the cause of altered flow times in the diseased eye.

## 4. Velocity

### 4.1. Blue Field Entoptic Technique

**Figure 4:**
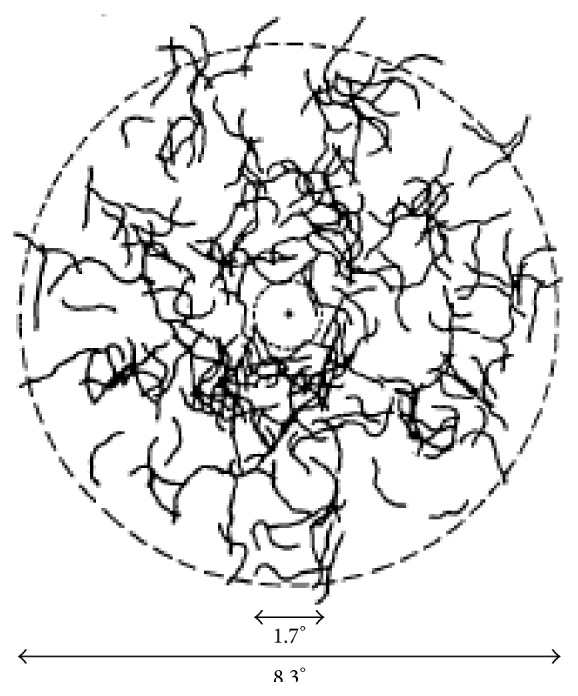
Simulation of retinal capillary paths along which the simulated leukocytes moved. These paths were not visible to the subjects. The cross at the center of the field is a fixation target. The angular diameters indicated in the figure correspond to viewing distance of 55 cm (reprinted from [46] Copyright Association for Research in Vision and Ophthalmology).

The blue field entoptic technique indirectly measures leucocyte speed in retinal perifoveal vessels [46]. With this technique, a participant is able to perceive their own leukocytes by looking into a uniform blue light at a wavelength of 430 nm. The participant then matches the speed and density of the observed leukocytes with the speed and density of computer simulated leukocytes (Figure 4) [46]. Measurements using this technique indicate that leucocyte speed in the capillaries is pulsatile, ranging from 0.5 to 1 mm/s. Participants are reliable in their ability to accurately match average and fast leucocyte speed but are less reliable with slow speeds. However, variability in blood flow speed between days was observed in subjects who had a high reliability and accuracy of measurements. This may suggest a real difference in leukocyte speed [46].

Because the blue field technique requires that blue light is shined on the retina, the effects of light stimulation on the macula required investigation. Qualitatively, participants observed an increase in leukocytes speed shortly after the flickering of blue light. Using an 8 Hz flickering light it was determined that leukocyte speed at the macula increases in less than 8 seconds of stimulation. The response is short-lived, lasting approximately 15 seconds before returning to baseline speed [47]. Since the blue field entoptic technique can be used to measure blood flow over the course of several minutes, it is unlikely that the blue light has a significant effect on blood flow results.

Blue field entoptic technique has been used to study vessel reactivity during changes in perfusion pressure. One investigation sought to measure the changes in flow during an artificial decrease in intraocular pressure (IOP) [48]. It was found that the max IOP in which regulation would occur and maintain a constant blood flow was 29.6 ± 2.0 mmHg. Beyond this level, the leukocyte speed decreased. Furthermore, the retina was able to compensate for a decrease of 36% in IOP [48]. This demonstrates a range of IOP at which vascular reactivity can effectively maintain a constant retinal circulation.

Retinal leukocyte speed and density during controlled exposures to oxygen and carbon dioxide have also been measured using blue field entopic technique [49]. Exposure to a mild hypoxic state (16% O_2_) did not alter leukocyte speed. However, exposure to pure oxygen caused a dramatic decrease in both leukocyte speed and density in the perifoveal capillaries. This response can be more than reversed by supplementing the pure oxygen with 5% CO_2_ causing an overall increase in leukocyte flow, despite the presence of excess oxygen [49].

The blue field entoptic phenomenon is an effective means of measuring both the leukocyte speed and density at the human macula. It is noninvasive and can be easily adapted to test flow during stimulation of the eye. However, this method is a subjective approach of estimating blood flow [46]. Accurate measurements heavily depended on participant cooperation and effectiveness at matching entoptic leukocyte speed and density to computer generated leukocytes. Because of this, investigations into vascular reactivity in the diseased eye are not practical.

### 4.2. Laser Doppler Velocimetry

Laser Doppler velocimetry (LDV) gives an absolute value for the maximum speed of red blood cells in large retinal vessels. This technique provides real time velocity monitoring in arteries and veins by calculating Doppler shifts in light scattered by flowing blood cells. When a low power coherent laser is positioned on a retinal vessel, the reflected laser light experiences a shift in frequency that is directly proportional to the flow velocity of the red blood cells. The maximum velocity corresponds to the maximum frequency shift located at the center of the vessel. LDV is commonly used in combination with previously mentioned techniques for determining vessel diameter. From cross-sectional area and velocity measurements, an estimated mean retinal blood flow can be calculated for a single vessel [50–52].

Using LDV, the effects of acute changes in IOP caused by scleral suction were investigated. By artificially increasing the IOP, a decrease in retinal perfusion pressure was induced. Riva et al. recorded that blood flow was effectively maintained if IOP did not exceed 27–30 mmHg and incomplete control was present at an IOP of 42 mmHg. The removal of the suction cup and consequent decrease in IOP resulted in a rapid increase in red blood cell velocity above initial baseline measurements. Velocity then returned to baseline within a few minutes [53, 54]. Another less invasive method of altering perfusion pressure is through experimental changes in systemic blood pressure. Robinson et al. employed isometric exercise to induce an acute rise in arterial blood pressure. They found that, in normotensive participants, there was no detectable change in blood flow, until a 41% increase in baseline blood pressure values [55]. At this point, vascular reactivity was ineffective, and blood flow increased in parallel with increases in blood pressure. Computer-assisted analysis of LDV images taken during isometric exercise demonstrated a similar response with an 8.4% increase in flow velocity after a 34% rise in perfusion pressure [56].

Since isometric exercise is an impractical and potentially dangerous means of increasing blood pressure in unhealthy participants, one study raised systemic blood pressure using injections of tyramine. In this study diabetics with high blood glucose failed to regulate flow during any increase in blood pressure [57]. This emphasizes the role of hypertension in the pathology of diabetic retinopathy. In another study conducted by Nagaoka et al., a cold pressor test was invoked to induce an acute arterial blood pressure increase of 15% when the hand was placed in 4°C water for 5 minutes. This method allowed for the simultaneous LDV analysis during stimuli that was not previously possible using an isometric exercise. Findings in this study suggest that the constriction of retinal arterioles plays an important role in regulating blood flow during acute increases in blood pressure [58, 59]. However, the cold pressor test can cause discomfort and may not be practical in a clinical setting [60].

**Figure 5:**
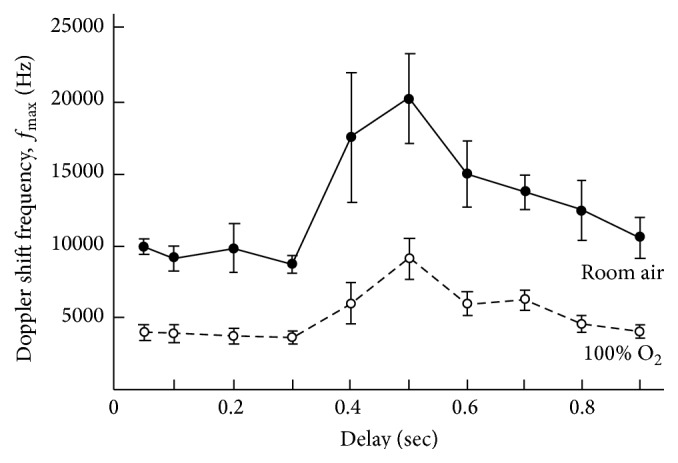
Variation of *f*_max_ and *v*_max_ during the cardiac cycle in an artery when breathing room air and after 5 min of 100% O_2_ breathing. The error bars represent ± standard deviation based on at least five measurements (reprinted from [61] Copyright Association for Research in Vision and Ophthalmology).

By combining the vessel diameter and maximum velocity measurements, LDV has been used to demonstrate the effects of breathing 100% oxygen on retinal blood flow (Figure 5) [61]. It was found that a 12% decrease in diameter and a 53% reduction in maximum velocity produced a 60% overall blood flow reduction [61]. This reduction in flow is in line with the 57% decrease in flow found by Hickam and Frayser using DDT [34]. Another study found that carbogen (95% oxygen, 5% carbon dioxide) slightly lessens the decreased retinal blood flow caused by breathing pure oxygen (42% reduction with carbogen, 56% reduction with oxygen) [62]. No significant difference of vascular reactivity in the temporal region (41.82% reduction in flow) and nasal region (36.05% reduction in flowing) during hyperoxia was found [63].

Laser Doppler velocimetry has been used to further confirm the hemodynamic deficits associated with diabetic retinopathy [64–67]. After 5 minutes of 100% oxygen, diabetic subjects without retinopathy showed slightly weaker control of blood flow compared to normal participants (53% reduction and 61% reduction, resp.). Subjects with both nonproliferative and proliferative diabetic retinopathy had a significantly smaller response (38% reduction and 24% reduction, resp.). However, after treatment with panretinal photocoagulation, proliferative diabetic retinopathy subjects improved in vascular response (54% reduction) to almost level of healthy subjects [66, 67].

Laser Doppler velocimetry is limited by its complexity. Alignment of the laser angle, optical fibers, and fixation target require a significant understanding of the instrument. Although LDV has provided valuable insight into vascular reactivity during various types of stimulation, the user experience required for this system makes the transition from research to clinical use difficult.

### 4.3. Laser Doppler Flowmetry

Laser Doppler Flowmetry (LDF) is a technique similar to LDV; however, instead of directing the laser light on retinal vessels, LDF measures blood flow at the optic nerve head (ONH) away from large, visible vessels. By applying the theory of Bonne and Nossal, light scattered from this tissue can be detected to determine the Doppler shift power spectrum caused by the in-depth flow of red blood cells [68]. The result is a relative measurement of mean velocity and volume of blood flow [68–70]. The LDF method, originally mounted on a fundus camera, could also be combined with a scanning laser tomography technique in order to provide a two-dimensional image of ONH and peripapillary retinal blood flow [71–73].

Laser Doppler Flowmetry has been extensively used to investigate vessel reactivity to stimuli in the animal eye [68, 69, 74, 75]. Recently, LDF has been adapted to the human eye in order to investigate reactivity during changes in blood gases, isometric exercise, and flickering light stimuli. Hyperoxia and hypercapnia have been shown to cause changes in blood flow similar to those recorded by previously mentioned techniques [76]. During hyperoxia, smokers were found to have less of a decrease in ONH blood flow (13%) compared to nonsmokers (37%) [77]. LDF has improved on previous blood gas investigations by demonstrating that vasoconstriction occurs before changes in capillary blood flow [77]. Furthermore, flow regulation during variations in IOP was found to occur by causing changes in blood volume and not blood velocity [78]. Lastly, LDF has shown increases in ONH blood flow in response to flickering light [79–82]. The luminance flicker-evoked response was reduced in patients with ocular hypertension and early glaucoma suggesting an impairment of vascular function [82].

A question that remains regarding LDF is the depth of the laser beam in the sampled tissue. In vivo, light penetration depth has been shown to be 300 *μ*m, although in vitro studies have demonstrated motion detection of microspheres in a glass capillary behind 600 *μ*m of excised ONH tissue [69, 78]. Another potential limitation is that LDF measurements can be affected if the beam spot is placed on a superficial vessel that is not large enough to be visible. However, automation of flow calculations and stabilization of laser beam spot on the retina are likely to improve the usefulness of LDF [83, 84].

### 4.4. Color Doppler Imaging

Originally developed for monitoring blood flow in the heart, color Doppler imaging (CDI) uses ultrasound to combine structural B-scans with velocity measurements determined from the Doppler shift of moving red blood cells [85, 86]. The mean flow velocity is then calculated from the peak systolic and end diastolic velocity [87]. The validity and reproducibility of CDI velocity measurements have been established with coefficients of variation of 4% for resistive index and 11% for peak systolic velocity [88, 89].

Using CDI, Kolodjaschna et al. describe a method of causing a rapid decrease in blood pressure by inflation and then rapid deflation of bilateral thigh cuffs [90]. This induces a decrease in systemic blood pressure (ranging between 9% and 15%) and subsequent acute decrease in ocular perfusion pressure. Blood pressure then returned to baseline 7 to 10 heart beat cycles later. Results of this study showed a difference in vascular response in the middle cerebral artery and ophthalmic artery [90]. Postural changes have also been used to induce acute changes in perfusion pressure. Changes in blood flow during supine and upright posture in healthy and glaucoma patients were studied using CDI [91]. Postural changes were found to expose a deficit in autoregulation among glaucoma patients, most prominently in vessels distal to the central retinal artery.

Limitations of CDI are inherent to the physics of Doppler ultrasound. Blood flow that is below the Doppler threshold or flow present in large tumors may not be determined due to poor Doppler angle or undetectable Doppler shifts [86]. Furthermore, pressure applied to the eyelid by CDI may cause a significant change in IOP resulting in changes in perfusion pressure [11]. Many studies have used CDI to investigate orbital hemodynamics in the diseased eye, although CDI has not been extensively used to study vascular reactivity during stimulation of the diseased eye [92–95].

### 4.5. Doppler Optical Coherence Tomography

Optical coherence tomography (OCT) has become the standard for structural imaging and evaluation of eye diseases [96, 97]. This is because of its ability to produce images of ocular anatomy with micron precision using a noninvasive and nonintrusive invisible infrared light. Traditional OCT provides structural images which enhance the clinician's ability to detect and monitor fluid exudation linked to vascular diseases. However, structural OCT is only sensitive to backscattered light intensity. An extension of this technology, called Doppler OCT (DOCT), allows for detection of the Doppler frequency shift caused by flowing red blood cells [98, 99]. A considerable improvement is that DOCT is capable of detecting volumetric blood flow together with structural anatomy. Several DOCT methods have been developed utilizing both time and Fourier domain that are sensitive to the shift in phase of the backscattered light [98–103].

The response of total retinal blood flow to flickering light was investigated using DOCT. Unlike previous studies, DOCT was capable of determining the response of the entire retinal circulation in absolute volumetric terms [104]. The total retinal blood flow was found to increase by an average of 22.2%. Furthermore, a significant increase in venous diameter but not arterial diameter was reported. This is different from previous studies, which showed increases in both veins and arteries [17]. The effects of hyperoxia studied using DOCT were found to be in good agreement with previous reports of velocity measured with LDV [105]. However, one study using the bilateral thigh occlusion cuffs technique mentioned earlier was not able to demonstrate regulation of inner ocular vessels [106].

One limitation with DOCT is the phase wrapping artifact that occurs in vessel with high blood flow velocities. However, this problem can be overcome by using faster cameras or swept source OCT [107–109]. Furthermore, Doppler OCT is only sensitive to blood flow that is parallel to the OCT beam.

## 5. Optical Coherence Tomography Angiography

OCT angiography is a significant breakthrough in ophthalmic imaging and may very soon change clinical practices [110]. OCT angiography has been described in some form since 2006 [111–113]. However, its application to ocular diseases has only begun to be explored in 2013 and the technology first became commercially available only in 2014 [114–118].

This technology does not require the injection of extrinsic contrast dye such as fluorescein or ICG. Instead, OCT angiography detects the motion of red blood cells as intrinsic contrast and therefore is sensitive to both transverse and axial flow in time [112, 113]. Cross-sectional OCT angiograms combine color-coded flow information superimposed on gray-scale structural information. Therefore, both blood flow and retinal structural information are presented together. This is useful for clinical evaluation on the depth of abnormalities. Since OCT angiography generates a data cube, segmentation and* en face* presentation of vascular perfusion at various layers of the retina can summarize the flow information at relevant anatomic layers or “slabs.” These images can be more easily interpreted by clinicians and aid in their ability to recognize abnormalities in vascular patterns such as capillary dropout or pathologic vessel growth of the outer retina and vitreous space.

Multiple approaches for OCT angiography have been developed. These include amplitude-based, phased-based, or combined amplitude/phase variance-based methods [119–123]. Furthermore, new software algorithms have been developed which allow existing OCT hardware to perform OCT angiography and use either the Doppler shift or variations in speckle pattern caused by moving red blood cells to detect both transverse and axial flow. These methods have become practical now because the high speed of Fourier-domain OCT allows multiple cross-sectional images to be obtained at the same location in very quick succession to detect relative motion in voxels containing blood flow [124–129]. Both varieties of Fourier-domain OCT, spectral (a.k.a. spectral-domain or spectrometer-based) or swept source, could be used [122–125]. Three-dimensional volumetric OCT angiography can be obtained in seconds.

**Figure 6:**
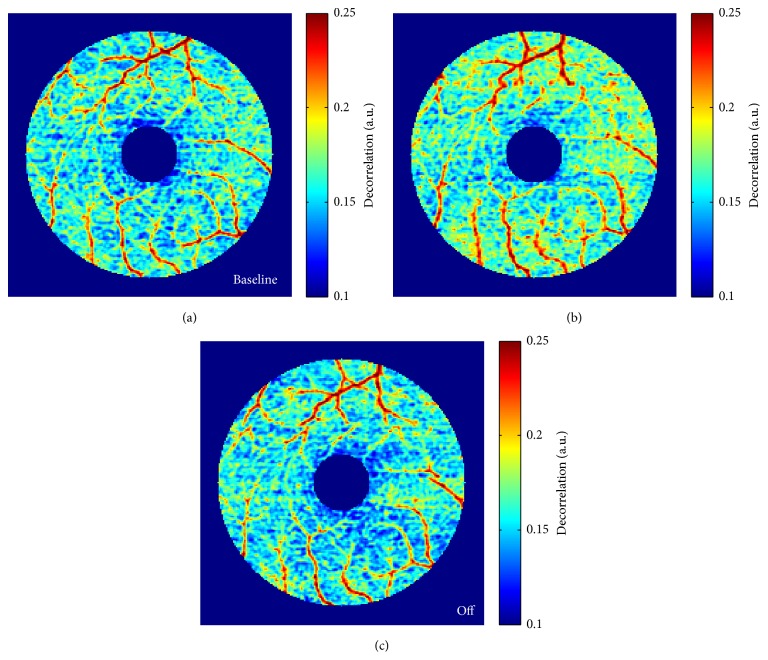
False color representation of* en face* retinal angiograms captured during the course of the experiment. Increased flow (warmer color, higher decorrelation values) was seen in the angiogram captured 30 seconds after the visual stimulation was turned on (b) compared to baseline (a). The angiogram captured 30 seconds after stimulation was turned off (c) did not appear different from baseline (reprinted from [133]).

Although OCT angiography has been used previously to detect retinal vessel reactivity in animals, only recently has this technology been applied to investigating stimulation of the human eye [130]. Developed by Jia et al. split-spectrum amplitude-decorrelation angiography or SSADA is an algorithm that is capable of flow detection both at the optic nerve head and at the macula and quantifies the data as both flow index and vessel density [131, 132]. Changes in these parameters have been detected during light flicker and hyperoxia stimulation. An 8 Hz reversing checkerboard pattern of light caused a maximum response of 7.2% increase in parafoveal retina flow index after 30–45 seconds (Figure 6) [133]. The flow index response was larger than previously measured vessel diameter increase but smaller than known changes in blood flow [133]. The pattern used here reduced luminance and provided constant illumination, allowing for 2 minutes of light stimulation, twice as long as previous experiments. However, saturation was found to occur and differences in flow index were not significant after 60 seconds of light flicker [133].

**Figure 7:**
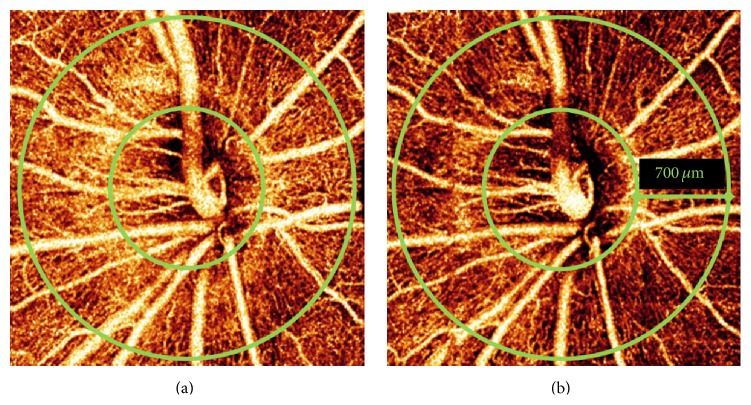
Optic disc* en face* maximum decorrelation projection angiograms from a participant with a large response to hyperoxia at baseline (a) and hyperoxia (b). The images represent a 3 × 3 mm area. The optic disc was delineated by the authors and is shown here with inner green ring. The outer green ring is defined as extending 700 *µ*m outward from inner disc. The image after hyperoxia exposure (b) shows a reported 17% decrease in flow index and a 4% decrease in vessel density (reprinted from [134]).

SSADA based OCT angiography has also been used to detect a decrease in peripapillary retinal blood flow in response to hyperoxia (Figure 7) [134]. During 60% supplemented oxygen, a decrease of 8.87 ± 3.09% (mean ± standard deviation) in flow index and 2.61 ± 1.50% in vessel density was observed. Overall, the flow index was found to be more sensitive than vessel density in detecting vascular reactivity. However, the relatively small response in flow index was only 2.4 times larger than the between-day standard deviation of 3.71%. Furthermore, the population variation in hyperoxic response was large, 36.9% (CV) for flow index and 57.5% (CV) for vessel density [134].

Because flow index and vessel density are determined from decorrelation values, the SSADA signal in large vessels with fast velocities becomes saturated. Flow index, therefore, does not provide a volumetric blood flow value and measurements are likely to underestimate actual blood flow changes. Because the OCT beam spot may be larger than the diameter of the capillaries, vessel density is likely to overestimate actual vessel density while underestimating change in size [134]. However, both flow index and vessel density measurements were shown to have good within-day repeatability and between-day reproducibility.

## 6. Discussion

This review paper describes an evolutionary process of the imaging technologies used for investigational ophthalmology of vascular reactivity. As the technology advanced, the parameters in which hemodynamics were detected became more sensitive to retinal blood flow, beginning with qualitative observations of vessel caliber made with the ophthalmoscope and advancing to 3D retinal angiograms provided by OCT angiography. Because of the different methods and locations in which these technologies detect retinal circulation, it is difficult to make direct comparisons between measurements made by various systems. However, the overall trends of vascular reactivity to direct stimulation of the eye or the effects of various physiological conditions can be compared across the evolution of imaging technology.

Vascular reactivity in response to simulation of the human eye has been well studied in the scientific literature. Characterization of the magnitude and time course of retinal blood flow response has led to a better understanding of the mechanism in which blood flow is regulated, as well as identifying functional impairments in the diseased eye. The data presented in the literature has made it clear that control of retinal circulation is a complex system and is dependent on the interactions of many metabolic factors and mechanisms that work together in order to provide adequate nutritional supply [11]. Considering this, it makes sense that although the experiments described in this review aim to isolate and study the effects of a single stimulus, it is likely that many other uncontrollable variables have a significant effect on vascular reactivity. This may explain the variability in response seen between days in a single healthy individual and within a healthy population. Slight variations in similar approaches to stimulating the eye also have a significant effect on vascular reactivity, despite the overall response trend remaining constant. Therefore, it is difficult to quantify and define a standard range in normal response to stimuli within a healthy population.

An important finding of the experiments described in this review is the characterization of vascular reactivity impairments in the diseased eye. Since light stimulation is currently the most noninvasive approach to stimuli and it can be adapted to existing hardware, it has the most potential for future use in the clinic. However, no technique is currently able to provide the highly sensitive and highly repeatable measurements needed to establish a diagnostic definition of vascular reactivity during light stimulation of the diseased eye. In the future, OCT angiography has great potential for achieving these requirements. One distinct advantage of this technique is that it can be applied as a software upgrade to OCT systems already in place. This would provide a large population in which the effects of stimulation could be established. A standardized response range of healthy ocular vessel reactivity could then be used to better characterize the impairments in diseased retina blood flow. Success of any eye stimulation technique or imaging modality is determined by its practicality for clinical use and its reliable detection of vascular reactivity response.
